# Wnt and Frizzled RNA expression in human mesenchymal and embryonic (H7) stem cells

**DOI:** 10.1186/1750-2187-3-16

**Published:** 2008-09-26

**Authors:** Ujunwa C Okoye, Craig C Malbon, Hsien-yu Wang

**Affiliations:** 1 Department of Pharmacology, Undergraduate Pharmacology Program, School of Medicine, State University of New York at Stony Brook, Stony Brook, NY 11794-8661, USA; 2 Department of Pharmacology, Diabetes & Metabolic Diseases Research Center, School of Medicine, State University of New York at Stony Brook, Stony Brook, NY 11794-8661, USA; 3 Department of Physiology & Biophysics, School of Medicine, State University of New York at Stony Brook, Stony Brook, NY 11794-8661, USA

## Abstract

**Background:**

Wnt signals are important for embryonic stem cells renewal, growth and differentiation. Although 19 Wnt, 10 Frizzled genes have been identified in mammals, their expression patterns in stem cells were largely unknown.

**Results:**

We conducted RNA expression profiling for the Wnt ligands, their cellular receptors "Frizzleds" and co-receptors LRP5/6 in human embryonic stem cells (H7), human bone marrow mesenchymal cells, as well as mouse totipotent F9 teratocarcinoma embryonal cells. Except failing to express Wnt2 gene, totipotent F9 cells expressed RNA for all other 18 Wnt genes as well as all 10 members of Frizzled gene family. H7 cells expressed RNA for each of the 19 Wnt genes. In contrast, human mesenchymal cells did not display detectable RNA expression of Wnt1, Wnt8a, Wnt8b, Wnt9b, Wnt10a, and Wnt11. Analysis of Frizzled RNAs in H7 and human mesechymal cells revealed expression of 9 members of the receptor gene family, except Frizzled8. Expression of the Frizzled co-receptor LRP5 and LRP6 genes were detected in all three cell lines. Human H7 and mouse F9 cells express nearly a full complement of both Wnts and Frizzleds genes. The human mesenchymal cells, in contrast, have lost the expression of six Wnt ligands, *i.e*. Wnt1, 8a, 8b, 9b, 10a and 11.

**Conclusion:**

Puripotent human H7 and mouse F9 embryonal cells express the genes for most of the Wnts and Frizzleds. In contrast, multipotent human mesenchymal cells are deficient in expression of Frizzled-8 and of 6 Wnt genes.

## Background

Wnt, a class of 19 secreted proteins in mammals, constitutes one of the most important families of signaling molecules that direct aspects of development, such as cell differentiation, cell fate determination as well as some processes in adult tissues [1-3]. Recently, Wnt signaling has been implicated in self-renewal of human and mouse embryonic stem cells [4-10], however, weather Wnts are expressed in these stem cells is largely not known. Wnt functioning as ligands operates via binding to products of the *frizzled (Fz) *gene family [11-13] which activates signaling pathways. Three Wnt signaling pathways have been elucidated, namely Wnt/β-catenin (canonical) pathway, Wnt/Ca^2+ ^(noncanonical) pathway and Wnt planar cell polarity (PCP) pathway [14]. It is the activation of Wnt canonical pathway implicated in stem cell renewal, at least in short-term experiments [9,15]. Weather or not other Wnt pathways are involved in stem cell renewal is not known. Activation of downstream heterotrimeric G-proteins and the novel phosphoprotein Dishevelleds (Dsh, Dvl1-3) by Frizzled upon binding of the cognate Wnt occurs in all Wnt pathways [16-21]. In Wnt canonical pathway, Gαq and phospholipase Cβ are activated. The activation of these enzymes leads to accumulation of intracellular inositol pentakisphophate which, in turn, represses the serine/threonine kinase glycogen synthase kinase-3β (GSK3β, *a.k.a. *zeste white3/shaggy in *Drosophila*) [22]. As GSK3 represses β-catenin, its inhibition in response to Wnt/Frizzled activation de-represses β-catenin (Armadillo in *Drosophila*) levels. Phosphorylation of β-catenin by GSK3β targets β-catenin for ubiquitination and proteosome-mediated degradation [23-25], which occurs within a large complex that involves Axin, the product of the *adenomatous polyposis coli *(APC) gene, protein phosphatase 2A, GSK3β, and β-TrCP [26,27]. Wnt activation of Frizzled and Dvl-mediated inhibition of GSK3 allows β-catenin to accumulate in the nucleus, interacting with members of the lymphoid enhancer factor (LEF)/T-cell factor (TCF) class of architectural high mobility group (HMG) box transcription factors [28-30]. Wnt1, Xwnt5, Wnt8 and other Wnts activate Wnt/β-catenin canonical pathway and induce duplication of the axis in Xenopus and Zebrafish. There is also growing evidence that some Wnts, like Xwnt4, 5a and 11, do not induce duplication of the axis, but rather cause morphogenetic defects in embryos, working through "Wnt/Ca^2+ ^pathway" that is distinct from the Wnt/β-catenin pathway described above [31].

All of the Frizzleds display a deduced protein sequence with the following properties: seven hydrophobic segments of sufficient length to be transmembrane-spanning domains [11,32].; an N-terminal region that is exofacial and N-glycosylated [32], a cytoplasmic C-terminal domain replete with sites suitable for possible phosphorylation [33], and overall homology to members of the superfamily of 7-transmembrane segment-containing receptors known to signal via heterotrimeric G-proteins [33,34]. In mouse embryonic carcinoma stem cells (F9), Wnt3a binds to Fz1 and leads to β-catenin accumulation and Lef/Tcf-dependent treanscriptional activation [17,35]. In Zebrafish embryos, signaling via rat Fz2, but not rat Fz1, has been shown to activate calcium transients, that appear by several criteria to mimic the responses of other GPCRs that activate the effector phospholipase Cβ (PLCβ), stimulate accumulation of water-soluble inositol phosphates and diacylglycerol, and ultimately activate protein kinase C [36,37]. Similarly, in frog oocytes activation of rat Fz-2, but not rat Fz-1, leads to PKC activation and recruitment of GFP-tagged PKC from the cytoplasm to the plasma membrane [38].

Wnt directly binds to the Cysteine-rich domain of Fz and the Wnt-Fz specificity determines Wnt signaling output. For Wnt-Fz signals via canonical pathway, recent work has identified low density lipoprotein receptor related protein (LRP) 5 or 6 (Arrow in Drosophola) as co-receptor of Wnt [39-41]. LRP5 and 6 are functionally redundant. Double homologous mutants of LRP5 and 6 fail to establish a primitive streak, the first morphological sign of gastrulation, mimicking mouse embryos which loss either β-catenin or Wnt3a [42].

Transcriptional profiling is used commonly to ascertain gene expression patterns by cells in culture, including human embryonic stem cells [43,44]. Since the Wnt-Frizzled signaling pathways are intimately associated with differentiation, cell fate, and early development, we conducted transcriptional profiling of the Wnt and Frizzled genes in three stem cells, *i.e.*, human embryonic (H7), human mesenchymal (hM), as well as mouse teratocarcinoma F9 embryonal (F9) cells. H7 and F9 cells are pluripotent, whereas hM are multipotent, *i.e. *hM cells are only capable of differentiation to lineages of mesenchymal tissues, such as bone, cartilage, fat, muscle and tendon. H7 cells expressed RNA for each of the 19 Wnt genes. Our data for human H7 cells and for mouse F9 cells suggest that these embryonal cells also express the genes for most of the Frizzleds. Surprisingly, the human mesenchymal cells were found to have lost the expression of Frizzled-8 and six Wnt genes.

## Results and discussion

**Figure 1:**
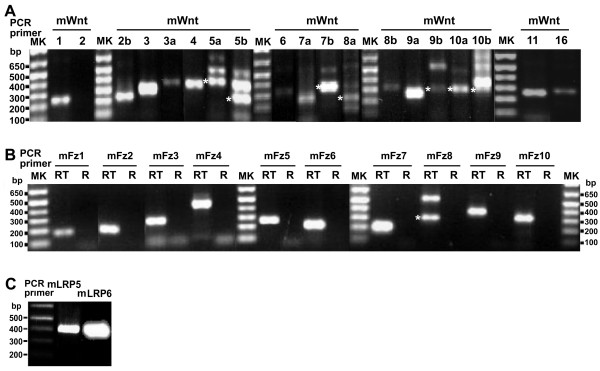
**Expression of Wnt and Frizzled genes in mouse totipotent teratocarcinoma F9 embryonal cells**. RNA was extracted from mouse F9 cells and then reverse-transcribed (RT) to the first strand cDNA. PCR amplification of the cDNA was conducted using specific primers for mouse Wnt1 through Wnt16, mouse Frizzled-1 through Frizzled-10 as well as for LRP5 and LRP6 (Tables 1, 2). The predicted sizes of the PCR products are listed in Tables 1 and 2. Data displayed are from one set of representative RT-PCR products of mouse F9 cells with primers specific for Wnts (A), Frizzleds (B), and LRP5/6 (C). R and RT in panel B indicate results of PCR performed on either the total RNA or the products of reverse transcription, respectively. Markers of DNA fragments (MK, bp) are provided and sizes labeled. In the case of appearance of multiple bands in panels A and B, each band of PCR product was purified and sequenced. * denoted the band corresponding to expected Wnt or Fz. Extraction of RNA were performed and subjected to RT-PCR using whole-cell preparations from at least three independent sources, each prepared on separate occasions.

The RT-PCR amplification was performed first on RNA extracted from mouse F9 embryonal cells. Primers were prepared to enable interrogation of all of the 19 Wnt genes (Table 1, Figure 1A). Base pair markers (100–650 bp, MK) are provided for the analysis of the PCR products. The results show the presence of mRNA for all Wnts except Wnt2. The RNA for Wnt2 was absent, even when performed under conditions that maximize detection of low-abundance RNAs (data not shown). Thus mouse F9 cells display expression of virtually all of the known Wnt genes except Wnt2. Primers were prepared also to enable PCR amplification of RNA encoding the cellular receptors for the Wnts, *i.e.*, the Frizzled family of G-protein-coupled receptors (Table 2, Figure 1B). PCR products obtained from the mouse F9 cells revealed RNA for all ten of the Frizzled family. Thus, mouse F9 cells do express nearly the full complement of Wnt and Frizzled genes. We interrogated whether or not the genes of co-receptors, *i. e. *LRP 5 and 6, obligate for canonical Wnt signaling, were expressed. Both LRP5 and LRP6 co-receptor genes are expressed in the mouse F9 cells (Table 2, Figure 1C), suggesting that with the exception of Wnt2, all of the proximal signaling ligands, receptors, and co-receptor genes are expressed in these cells.

**Table 1:** Primer sequences for RT-PCR analysis of mouse Wnt (mWnt) genes

**Gene**	**Accession No.**	**Sequence (5' to 3')**	**Amplicon (bp)**
mWnt1	NM_021279	(F) ccgagaaacagcgttcatct (R) gcctcgttgttgtgaaggtt	252
mWnt2	NM_023653	(F) Atctcttcagctggcgttgt (R) agccagcatgtcctcagagt	326
mWnt2b	NM_009520	(F) cacccggactgatcttgtct (R) tgtttctgcactccttgcac	236
mWnt3	NM_009521	(F) cgctcagctatgaacaagca (R) aaagttgggggagttctcgt	303
mWnt3a	NM_009522	(F) cccaacttctgcgaacctaa (R) tctccgccctcaagtaagaa	347
mWnt4	NM_009523	(F) aacggaaccttgaggtgatg (R) ggacgtccacaaaggactgt	345
mWnt5a	NM_009524	(F) ctggcaggactttctcaagg (R) ctctagcgtccacgaactcc	388
mWnt5b	NM_009525	(F) gggaccgtttgaaggagaag (R) ctcttgaagcggtcatagcc	259
mWnt6	NM_009526	(F) tcagttccagttccgtttcc (R) ttgacttctcatccccgaag	323
mWnt7a	NM_009527	(F) cgagagctaggctacgtgct (R) acagcacatgaggtcacagc	264
mWnt7b	NM_009528	(F) tacctaagttccgcgaggtg (R) acgtgttgcacttgacgaag	363
mWnt8a	NM_009290	(F) tgtcatggcatctcaggaag (R) gcggttgcagtagtcaggag	240
mWnt8b	NM_011720	(F) gtggacttcgaagcgctaac (R) ttacacgtgcgtttcatggt	316
mWnt9a	NM_139298	(F) tgctttcctctacgccatct (R) tatcaccttcacacccacga	256
mWnt9b	NM_011719	(F) aggagacggccttcctgtat (R) gcacttgcaggttgttctca	296
mWnt10a	NM_009518	(F) gcgctctgggtaaactgaag (R) cacacggttgttgtggagtc	302
mWnt10b	NM_011718	(F) ggaagggtagtggtgagcaa (R) cacttccgcttcaggttttc	298
mWnt11	NM_009519	(F) gtgcggacaacctcagctac (R) gacaggtagcgggtcttgag	259
mWnt16	NM_053116	(F) ctgtgaaaccaccttgcaga (R) caggttttcacagcacagga	261

**Table 2:** Primer sequences for RT-PCR analysis of mouse Frizzled (mFzd) and LRP genes

**Gene**	**Accession No.**	**Sequence (5' to 3')**	**Amplicon (bp)**
mFz1	NM_021457	(F) caaggtttacgggctcatgt (R) tgaacagccggacaggaaaa	178
mFz2	NM_020510	(F) ccgacggctctatgttcttc (R) tagcagccggacagaaagat	173
mFz3	NM_021458	(F) tgggttggaagcaaaaagac (R) cctgctttgcttctttggtc	239
mFz4	NM_008055	(F) gccaatgtgcacagagaaga (R) aggtggtggagatgaagcag	398
mFz5	NM_022721	(F) ctgtggtctgtgctgtgctt (R) ggccatgccaaagaaataga	264
mFz6	NM_008056	(F) tctgtgcctctgcgtatttg (R) tctcccaggtgatcctgttc	218
mFz7	NM_008057	(F) gcttcctaggtgagcgtgac (R) aacccgacaggaagatgatg	216
mFz8	NM_008058	(F) ttacatgcccaaccagttca (R)cggttgtagtccatgcacag	306
mFz9	NM_284144	(F) agtttcctcctgaccggttt (R) ttttcggtagcacaggctct	390
mFz10	NM_175284	(F) agattcccatgtgcaaggac (R) agttggggtcgttcttgttg	321
mLRP5	NM_008513	(F) Aagaccctgcttgaggacaa (R) gagtgggatagccacatcgt	402
mLRP6	NM_008514	(F) Gagctcatcggtgacatgaa (R) gctcgaggactgtcaaggtc	400

**Figure 2:**
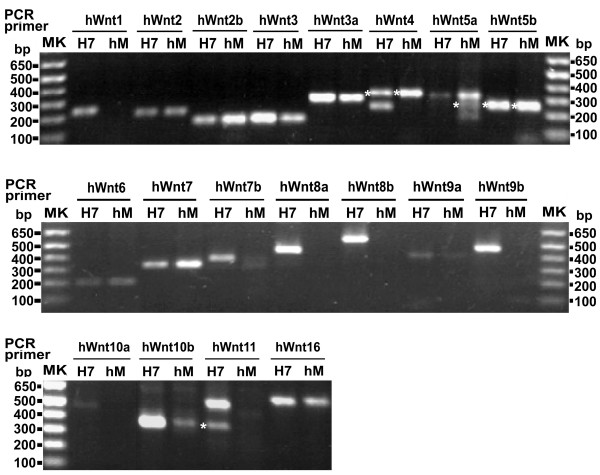
**Expression of Wnt genes in human embryonic H7 stem cells and human mesenchymal cells**. RNA was extracted from either human embryonic (H7) or mesenchymal (hM) cells and then subjected to reverse transcription to the first strand cDNA. PCR amplification of the cDNA was conducted using specific primers for human Wnt1 through Wnt16 (Table 3). The predicted size of the PCR products for each Wnt is listed in Table 3. The data displayed are from a representative PCR performed on the same whole-cell RNA and first subjected to RT. Markers of DNA fragments (MK, bp) are provided and the sizes labeled. Each separated band of PCR products was purified and subjected to DNA sequencing. Each band has been confirmed as expected gene products based upon DNA sequencing. * denoted a possible splicing form of corresponding genes. Extraction of RNA were performed and subjected to RT-PCR using whole-cell preparations from at least three independent sources, each prepared on separate occasions.

**Figure 3:**
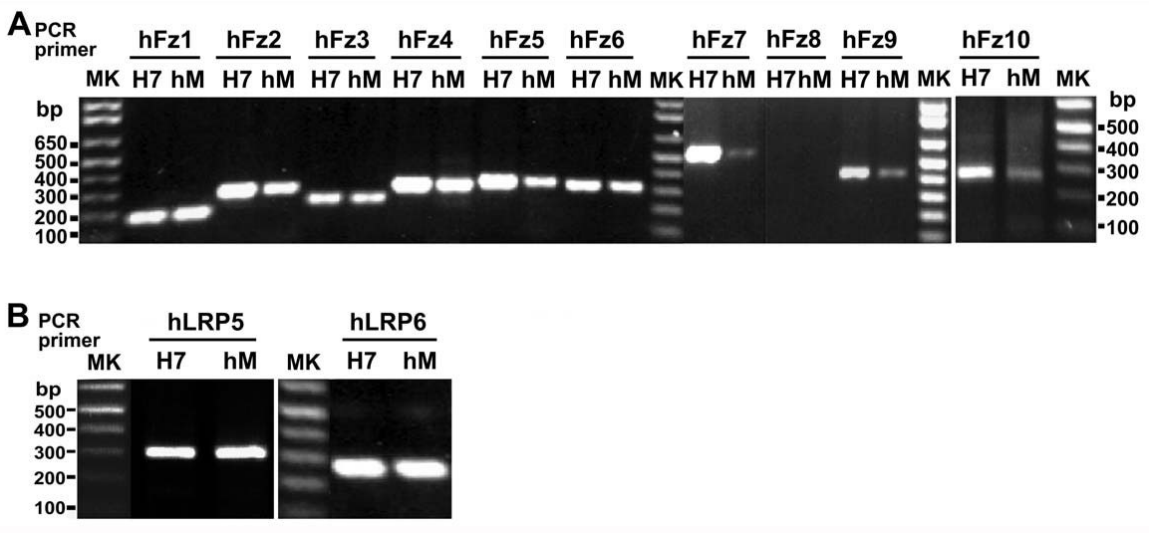
**Expression of Frizzled and LRP5/6 genes in human embryonic H7 stem cells or human mesenchymal cells**. RNA was extracted from either human embryonic (H7) or mesenchymal (hM) cells and then subjected to reverse transcription to the first strand cDNA. PCR amplification of the cDNA was conducted using specific primers for human Frizzled-1 through Frizzled-10 (A) or LRP5 versus LRP6 (B). The nucleotide sequence of each primer and predicted size of the PCR products for each Frizzled is listed in Table 4. The data displayed are from a representative PCR performed on the same whole-cell RNA and first subjected to RT. Markers of DNA fragments (MK, bp) are provided and the sizes labeled. Extraction of RNA were performed and subjected to RT-PCR using whole-cell preparations from at least three independent sources, each prepared on separate occasions.

We next interrogated whole-cell RNA extracted from two human stem cell lines, embryonic (H7) and mesenchymal (hM), by RT-PCR amplification using primers (Tables 3 and 4) designed to detect expression of all 19 members of the Wnt gene family (Figure 2), LRP5/6, as well as 10 members of the Frizzled gene family (Figure 3). The amplification products reveal that for embryonic H7 stem cells all 19 members of the Wnt gene family are expressed. These data contrast with the results of the RT-PCR amplification of the human mesenchymal cell RNA, in which six Wnt genes could not be detected, *i.e.*, Wnt1, Wnt8a, Wnt8b, Wnt9b, Wnt10a, and Wnt11. These data suggest that the more highly differentiated mesenchymal stem cells have lost the full complement of Wnt gene expression (Table 5). Thus human embryonic H7 stem cells display expression of the full complement of Wnt genes.

**Table 3:** Primer sequences for RT-PCR analysis of human Wnt (hWnt) genes

**Gene**	**Accession No.**	**Sequence (5' to 3')**	**Amplicon (bp)**
hWnt1	NM_005430	(F) gcgtctgatacgccaaaatc (R) ggattcgatggaaccttctg	244
hWnt2	NM_003391	(F) tagtcgggaatctgcctttg (R) ttcctttcctttgcatccac	221
hWnt2b	NM_004185	(F) ctcatcagcaggggtagtcc (R) aaaacggacaccgtagtgga	159
hWnt3	NM_030753	(F) acgagaactcccccaacttt (R) gatgcagtggcatttttcct	170
hWnt3a	NM_033131	(F) tgttgggccacagtattcct (R) atgagcgtgtcactgcaaag	302
hWnt4	NM_030761	(F) ccttcgtgtacgccatctct (R) gcctcattgttgtggaggtt	250
hWnt5a	NM_003392	(F) ccacatgcagtacatcggag (R) cactctcgtaggagcccttg	378
hWnt5b	NM_032642	(F) gtgcagagacccgagatgtt (R) caggctacgtctgccatctt	550
hWnt6	NM_006522	(F) ggttatggaccctaccagca (R) aatgtcctgttgcaggatgc	208
hWnt7a	NM_004625	(F) agtacaacgaggccgttcac (R) gcacgtgttgcacttgacat	326
hWnt7b	NM_058238	(F) aagctcggagcactgtcatc (R) ccctcggcttggttgtagta	374
hWnt8a	NM_058244	(F) tggggaacctgtttatgctc (R) ccctcggcttggttgtagta	456
hWnt8b	NM_003393	(F) ctggtccaaaggcttacctg (R) tgagtgctgcgtggacttc	557
hWnt9a	NM_003395	(F) gacggtcaagcaaggatctg (R) tgctctcgcagttcttctca	411
hWnt9b	NM_003396	(F) ctgcttgagtgccagtttca (R) cgagtcatagcgcagtttca	477
hWnt10a	NM_025216	(F) aatgccaacaccaattcagg (R) caactcggttgttgtgaagc	464
hWnt10b	NM_003394	(F) gcaagagtttcccccactct (R) gattgcggttgtgggtatc	367
hWnt11	NM_004626	(F) ttgcttgacctggagagagg (R) gacgagttccgagtccttca	521
hWnt16	NM_007168	(F) tgctccgatgatgtccagta (R) acctcctgcaacggacatag	562

**Table 4:** Primer sequences for RT-PCR analysis of human Frizzled (hFz) and LRP genes

**Gene**	**Accession No.**	**Sequence (5' to 3')**	**Amplicon (bp)**
hFz1	NM_003505	(F) gtgagccgaccaaggtgtat (R) cagccggacaagaagatgat	184
hFz2	NM_001466	(F) gcgtcttctccgtgctctac (R) ctgttggtgaggcgagtgta	286
hFz3	NM_017412	(F) tgagtgttcgaagctctatgg (R) atcacgcacatgcagaaaag	229
hFz4	NM_012193	(F) aacctcggctacaacgtgac (R) gttgtggtcgttctgtggtg	303
hFz5	NM_003468	(F) aacctcggctacaacgtgac (R) gttgtggtcgttctgtggtgc	322
hFz6	NM_003506	(F) attttggtgtccaaggcatc (R) tattgcaggctgtgctatcg	311
hFz7	NM_003507	(F) gtgcagtgttctcccgaact (R) gaacggtaaagagcgtcgag	544
hFz8	NM_031866	(F) tcttgtcgctcacatggttc (R) tgtagagcacggtgaacagg	375
hFz9	NM_003508	(F) cgctggtcttcctactgctc (R) agaagaccccgatcttgacc	414
hFz10	NM_007197	(F) gcggtgaagaccatcctg (R) gcacggtgtacagcacagag	276
hLRP5	NM_002335	(F) accggaaccacgtcacag (R) gggtggataggggtctgagt	305
hLRP6	NM_002336	(F) aggcacttacttccctgcaa (R) gggcacaggttctgaatcat	274

**Table 5:** Expression of Wnt gene in mouse totipotent teratocarcinoma embryonic cell (F9), human embryonic stem cell (H7), and human mesenchymal cells (hM).

**gene\cell**	**F9**	**H7**	**hM**
**Wnt 1**	+	+	-
**Wnt 2**	-	+	+
**Wnt 2b**	+	+	+
**Wnt 3**	+	+	+
**Wnt 3a**	+	+	+
**Wnt 4**	+	+	+
**Wnt 5a**	+	+	+
**Wnt 5b**	+	+	+
**Wnt 6**	+	+	+
**Wnt 7a**	+	+	+
**Wnt 7b**	+	+	+
**Wnt 8a**	+	+	-
**Wnt 8b**	+	+	-
**Wnt 9a**	+	+	+
**Wnt 9b**	+	+	-
**Wnt 10a**	+	+	-
**Wnt 10b**	+	+	+
**Wnt 11**	+	+	-
**Wnt 16**	+	+	+

RNA was extracted from F9, H7 and hM and reverse-transcribed to obtain first strand cDNA. Specific primers were used in PCR to amplify each Wnt cDNA. Amplified products were purified and confirmed by DNA sequencing.

In our analysis of Wnt genes in human embryonic as well as mesenchymal stem cells we observed a few instances in which heterogeneity in the PCR products was observed in amplification reactions using the same pair of primers (Table 3, Figure 2). RT-PCR products for human Wnt4, Wnt5a and Wnt11 genes consistently yield PCR products of multiple sizes. We also observed that the size of PCR product for Wnt5b detected was much smaller than it was predicted (550bp). We extracted and purified PCR products from each separated band and subjected these samples to DNA sequencing. Results from DNA sequencing (data not shown) suggest the possibility that hWnt4, hWnt5a hWnt5b and hWnt11 gene products may undergo RNA splicing/editing. To our knowledge, this report is the first to make this novel observation.

We probed for expression for all members of the Frizzled gene family employing RNA extracted from H7 and hM cells for RT-PCR amplification. The amplification products revealed expression of all Frizzled genes, with the exception of that for Frizzled-8 (Figure 3A). PCR products for Frizzled-8 were not detected in reactions performed with either human cell source (Table 6). The gene for Frizzled-8 maps to human chromosome 10p11.2 and its expression has been shown to be amplified among human cancer cell lines [45]. Attempts to detect expression of the Frizzled-8 gene in either H7 or hM cell RNA preparations by RT-PCR with two other pairs of primers likewise failed to produce a product for this Frizzled gene (data not shown). Although detected in a variety of adult human cells and tissues [45], Frizzled-8 RNA appears to be undetected in RT-PCR amplifications prepared from whole-cell RNA isolated from both stem-cell sources, H7, and mesenchymal cells. Both LRP5 and LRP6 are expressed in human embryonic H7 and mesenchymal stem cells (Figure 3B).

**Table 6:** Expression of Frizzled and LRP genes in mouse totipotent teratocarcinoma embryonic cell (F9), human embryonic stem cell (H7), and human mesenchymal cells (hM).

**gene\cell**	**F9**	**H7**	**hM**
**Fz 1**	+	+	+
**Fz 2**	+	+	+
**Fz 3**	+	+	+
**Fz 4**	+	+	+
**Fz 5**	+	+	+
**Fz 6**	+	+	+
**Fz 7**	+	+	+
**Fz 8**	+	-	-
**Fz 9**	+	+	+
**Fz 10**	+	+	+
**LRP5**	+	+	+
**LRP6**	+	+	+

RNA was extracted from F9, H7 and hM and reverse-transcribed to obtain first strand cDNA. Specific primers were used in PCR to amplify each Wnt cDNA. Amplified products were purified and confirmed by DNA sequencing.

Taken together, our results suggest that embryonic stem (human H7 and mouse F9) cells express nearly a full complement of Wnts, their receptors, the Frizzleds, as well as their co-receptors, LRP5 and 6. The human mesenchymal stem cells, in contrast, have lost the expression of six Wnt ligands, *i.e*. Wnt1, 8a, 8b, 9b, 10a and 11. The absence of detection of RNA for Frizzled-8 in the human stem cell source, but not for the mouse F9 cells, is of interest and like the observation of possible RNA splicing of human Wnt4, Wnt5a, Wnt5b and Wnt11, deserving of further detailed analysis.

## Conclusion

Our data for pluripotent human H7 cells and mouse F9 cells suggest that these embryonal cells express the genes for most of the Wnts and Frizzleds. Multipotent human mesenchymal cells, in contrast, were found to be deficient in expression of Frizzled-8 and of six Wnt genes.

## Methods

### Materials

Mouse F9 teratocarcinoma cells were obtained from ATCC (Manassas, VA). Human embryonic H7 stem cells were obtained from WiCell Research Institute (Madison, WI). Human mesenchymal stem cells were purchased from Cambrex Bio Science (Walkersville, MD). Dulbecco's modified Eagle's medium (DMEM), Knockout serum, basic fibroblast growth factor, L-glutamine, penicillin and Streptomycin were from Invitrogen (Carlsbad, CA). Fetal calf serum was purchased from Hyclone (South Logan, UT). Matrigel was obtained from BD Bioscience (San Jose, CA). All primers employed in this study were synthesized by Operon (Huntsville, AL).

### Cell culture

Mouse totipotent teratocarcinoma F9 clones (ATCC,) were grown in DMEM supplemented with 15% heat-inactivated fetal calf serum at 37°C in a 5% CO_2_, 95% air incubator. The human mesenchymal stem cells were characterized by Lonza Bioscience (Walkersville, MD) for mesenchymal markers (CD29, 96% test positive; CD44, 97% test positive; CD105, 99% test positive; and CD 166, 95% test positive), macrophage marker (CD14, 97% test negative), as well as hematopoietic markers (CD34, 98% test negative; CD45, 99% test negative). The human mesenchymal stem cells were cultured and propagated according to the protocol provided by Cambrex Bio Science. Briefly, cells were cultured in tissue culture plates and maintained at 37°C in humidified 5% CO_2 _in mesenchymal stem cell growth medium (MSCGM) supplemented with L-glutamine, penicillin, streptomycin, and serum (MSCGM Bullet kit; Cambrex Bio Science). Passages of p3 to p5 were used for extraction of total RNA. Human embryonic stem cells (H7) were cultured on a layer of mouse embryonic fibroblasts, inactivated by mitomycin-C, in DMEM medium supplemented with 20% Knockout serum replacement, 0.1 mM nonessential amino acids, 2 mM L-GlutaMAX™, 1 mM sodium pyruvate, 0.1 mM β-mercaptoethanol, 4 ng/ml basic fibroblast growth factor, 50 u/ml penicillin and 50 u/ml streptomycin. H7 cells were cultured at 37°C in a 5% CO_2_, 95% air incubator. To prepare H7 cells free from MEF for RNA extraction, cells were rinsed once with Hank's buffered salt solution and incubated in DMEM containing 1 mg/ml dispase at 37°C for 30 min. Colonies dethatched from the culture plate were collected, rinsed off from dispase and gently dispersed into smaller pieces of cell aggregates by used a 5 ml pipette. Cell aggregates were placed in Matrigel coated plates, cultured for 2–4 days, and then RNA was extracted.

### Reverse transcription-polymerase chain reaction (RT-PCR) amplification

Total RNA was extracted by using STAT-60 kit from TEL-TEST (Friendswood, TX) according to the manufacturer's protocols. First strand cDNA was synthesized from 1 μg of total RNA by using random hexamer primers and Superscript II reverse transcriptase (Invitrogen). The first strand cDNA (0.1 μg) was subjected to polymerase chain reaction (PCR) (94°C for 45 s, 60°C for 45 s, 72°C for 1 min; for 35 cycles with a final extension at 72°C for 5 min) by using Taq DNA polymerase (Invitrogen) in tandem with specific primer pairs targeting each *Wnt, Frizzled *and *LRP *genes. Primer sequences are presented in Tables 1, 2, 3, 4. Samples from PCR reactions were separated electrophoretically on 0.8–1.0% agarose gels containing 0.2 μg/ml of ethidium bromide. Fluorescent bands of amplified gene products were captured by using GelDoc-It system (UVP LLC, Upland, CA).

### DNA sequencing

PCR products in agarose gels were isolated using the QIAquick Gel Extraction Kit from QIAGEN (Valencia, CA). Purified samples and correspondent PCR primers were submitted to the DNA sequencing core facility in the University campus.

## Abbreviations

Fz: Frizzled; LRP: low density lipoprotein receptor related protein; GPCR: G-protein coupled receptor; GSK3β: glycogen synthase kinase-3β; PLCβ: phospholipase Cβ; LEF: lymphoid enhancer factor; TCF: T-cell factor; hM: human mesenchymal.

## Competing interests

The authors declare that they have no competing interests.

## Authors' contributions

UCO carried out all experiments and analyzed data. CCM participated in coordination and helped to draft the manuscript. HYW designed the study, analyze data and drafted the manuscript. All authors read and approved the final manuscript.
